# The potential for using a Universal Medication Schedule (UMS) to improve adherence in patients taking multiple medications in the UK: a qualitative evaluation

**DOI:** 10.1186/s12913-015-0749-8

**Published:** 2015-03-11

**Authors:** Cassandra Kenning, Joanne Protheroe, Nicola Gray, Darren Ashcroft, Peter Bower

**Affiliations:** NIHR School for Primary Care Research, Centre for Primary Care, Manchester Academic Health Science Centre (MAHSC), University of Manchester, 5th floor, Williamson Building, Oxford Road, M13 9PL Manchester, UK; Institute of Primary Care and Health Sciences, Keele University, Keele, UK; Independent Pharmacist Researcher and Director- Green Line Consulting Ltd, Manchester, UK; Centre for Pharmacoepidemiology and Drug Safety Research, Manchester Pharmacy School, NIHR Greater Manchester Primary Care Patient Safety Translational Research Centre, University of Manchester, Manchester, UK; NIHR Greater Manchester Primary Care Patient Safety Translational Research Centre, Centre for Primary Care, Manchester Academic Health Science Centre (MAHSC), University of Manchester, Manchester, UK

**Keywords:** Medication adherence, Multimorbidity, Comorbidity, Reminder charts, Primary care, Pharmacy

## Abstract

**Background:**

Poor adherence to prescribed medication has major consequences. Managing multiple long-term conditions often involves polypharmacy, potentially increasing complexity and the possibility of poor adherence. As a result of the globally recognised problems in supporting adherence to medication, some researchers have proposed the use of reminder charts. The main aim of the research was to explore the need for and perceptions around the ‘Universal Medication Schedule’ (UMS). Looking at ways in which pharmacists and General Practitioners (GPs) could use the UMS in NHS settings.

**Methods:**

Semi-structured interviews were carried out with 10 GPs, 10 community pharmacists and 15 patients. Patients were aged 65 years and over, had multiple long-term conditions and were prescribed at least 5 medications. Interviews were recorded and transcribed and thematic analysis was conducted, using a framework approach to manage the data.

**Results:**

Attitudes towards the UMS were mixed with stakeholders seeing benefits and limitations to the chart. Practitioners proposed a number of existing services where they thought the UMS could easily be integrated but there was evidence of role conflict with GPs feeling it may be best placed with pharmacists and vice versa. The potential for the UMS to be used as a tool to aid communication between the different services involved in a patient’s care was a key theme.

**Conclusions:**

The UMS chart provides consolidated medicines information that might help to improve patients’ knowledge and health literacy, which may or may not improve adherence but could help patients in making informed decisions about their treatment. One of the key benefits of using the UMS in practice is that it could be introduced across services. In this way it may aid in medicines reconciliation between healthcare settings to ensure continuity of message, improve patient experience and create more joined up working between services. Further research is needed to test implementation in different services and to assess outcomes on patient understanding and adherence.

**Electronic supplementary material:**

The online version of this article (doi:10.1186/s12913-015-0749-8) contains supplementary material, which is available to authorized users.

## Background

Poor adherence to prescribed medication has significant consequences. It is estimated that 5-8% of hospital admissions in the UK are related to ineffective or inappropriate use of medicines [1]. A Cochrane review concluded that improving patients’ use of prescribed medicines may have a far greater impact on clinical outcomes than an improvement in treatments [2]. It has been estimated that £100 million per annum is wasted on unused prescription medicines in primary and community care in the UK [3].

Issues related to poor adherence may be particularly important in patients with multimorbidity. Multimorbidity is now common among patients with long-term conditions [4] and many of these patients require multiple medications. Polypharmacy, the prescribing of multiple medications to the same individual, increases the risk of adverse drug events [5,6], and increases the complexity of a regimen and thus the possibility of non-adherence [7]. Non-adherence may be intentional or unintentional [8]. Reasons for unintentional non-adherence may include difficulty opening medicine containers, lack of understanding of the regimen, or forgetting. Underlying reasons for intentional non-adherence will centre upon patient beliefs about their medicines and conditions. Poor health literacy may also contribute to either form of non-adherence [9]. It is thought that in the UK between a half and a third of all medicines prescribed for long-term conditions are not taken as recommended [10]. Research has shown that 90% of multi-medication prescriptions can be dosed four or fewer times a day [11]. However, some research has also shown that without explicit consolidation of medicines, patients tend to make their regimens more complicated and burdensome than is necessary. In a study conducted in the US, 464 patients were given a hypothetical 7-drug medication regimen and asked to demonstrate how and when they would take them, over a 24 hour period, according to the information given on the labels. On average patients identified 6 times (±1.8) in 24 hours to take the 7 medicines. One third dosed the medicines 7 or more times a day but only 15% stated 4 or fewer times a day (the ideal dosing schedule) [12].

As a result of the globally recognised problems in supporting patients to correctly adhere to their prescribed medication, some researchers have worked on the development of reminder charts. An early trial conducted in the UK assessed the impact of a reminder chart on patients understanding and adherence to medicine regimens on discharge from hospital. The charts were trialled with 197 patients on discharge from general medical wards who were prescribed between 2–6 medicines. The results showed that the reminder chart significantly improved patients’ knowledge of their medicines and also their adherence [13]. In the US, the ‘Universal Medication Schedule’ (UMS) has been developed [12]. Adherence is a complex and multifactorial issue and there is evidence that simplification of regimen alone does not solve the adherence problem [10]. Likewise just providing clear instructions/information is unlikely to improve adherence [14]. The UMS study aimed to try and address both these issues. The aim was to standardise prescription labelling and to provide a simple chart bringing all medicines in a patients’ regimen together over 4 dosing periods through the day and which also explains the purpose of each medication to improve understanding. The authors report that the UMS has demonstrated efficacy and effectiveness on comprehension, consolidation of regimens and improved adherence measured by pill count [12].

The UMS has been developed for use in the US care system and has not been evaluated as an intervention on its own, only in conjunction with simplification of prescription labels. Therefore, it was not clear if the results would be generalizable, feasible or acceptable to primary care practice in the UK. The aim of this study was to examine the perceived need for further support and to explore GP, pharmacist and patient attitudes towards the UMS chart and its potential for implementation in primary care.

## Methods

A qualitative study was undertaken in which semi-structured interviews were carried out with key stakeholders in patient management of medications. Ten general practitioners, 10 community pharmacists and 15 patients from the Greater Manchester area took part in interviews. GPs and pharmacists were recruited by the researcher and with help from the Greater Manchester Comprehensive Local Research Network (GMCLRN). After their interview GPs and pharmacists were each asked to identify up to 5 patients aged 65 years or over; with a vascular condition comorbid with at least 1 other long-term condition; and who had been prescribed at least 5 regular medications. Vascular diseases were selected to align with the project funder’s priorities (GM CLAHRC), and because GPs and community pharmacists have a primary role in supporting medicines use with these patients. Practitioners were asked to give information packs to patients inviting them to take part in an interview. If interested, patients were advised to return the reply slip with their contact details in the envelope provided. Patients were then contacted by the researcher to check eligibility and to arrange a time and date for the interview.

The patient sample consisted of 8 females and 7 males, with a mean age of 74.5 years (range 65–89 years). Patients had a mean of 4.2 conditions (range 2–7) and were prescribed a mean of 8.7 medicines (range 5–16). Table 1 shows the individual characteristics of the patients. The GP sample consisted of 6 males and 4 females from a range of general practices in Greater Manchester. Pharmacists were recruited from a range of companies: 4 from a nationwide multiple (>100 sites), 2 from regional multiples (>50 sites), 3 from regional groups (>10 sites) and 1 independent pharmacy.

**Table 1:** **Patient sample characteristics: gender, age, self-report long-term conditions and number of medications prescribed**

**ID No.**	**Gender**	**Age (years)**	**Conditions**	**Number of medications**
P1	F	67	CHD, Angina, Hypertension, Cholesterol	7
P2	F	81	DM II, Arthritis, Stroke, Depression, hypertension, Glaucoma, underactive thyroid	15
P3	M	81	Angina, COPD	6
P4	M	78	Heart condition, Stroke, Arthritis, blood clot in leg	8
P5	F	66	Heart condition, arthritis	7
P6	M	65	Malignant hypertension, kidney disease, complex regional pain disorder, prolapse disc	14
P7	M	73	Depression, heart attack, arthritis, angina, hypertension	11
P8	M	73	Heart condition, hypertension, cholesterol, severe pain following polio	6
P9	M	74	Ischemic heart disease, hypertension, cholesterol, blind in right eye, cataract left eye	6
P10	F	82	TIA, Angina, COPD, DM II, cellulitis, hypertension	11
P11	F	82	Osteoporosis, heart condition, arthritis, COPD	16
P12	F	66	TIA, hypertension, COPD, cholesterol	5
P13	F	71	Angina, hypertension, arthritis, asthma	8
P14	M	89	CHD, COPD, osteoporosis, angioneurotic oedema	6
P15	M	69	Heart attack, DM II, hypertension, cholesterol	5

Practitioner interviews focussed on exploring their thoughts on patient understanding and adherence to their medication, barriers to - and the importance of - good understanding as it related to both unintentional and intentional non-adherence. The interviews also explored views on current practices and services to help understanding and adherence, and their views on the UMS (see Table 2 for the example chart) including contents/format of the chart, which patients it could be useful for, how it might be linked into current practice, and any barriers or limitations they foresaw in its implementation (See Additional file 1- practitioner interview schedule).

**Table 2:** **Example Universal Medication Schedule (UMS) chart**

**Name of medicine**	**Breakfast**	**Lunchtime**	**Evening Meal**	**Bedtime**	**Special instructions**
**Amlodipine 5 mg**	1 tablet				For blood pressure
**Lisinopril 10 mg**	1 tablet				For blood pressure
**Simvastatin 20 mg**				1 tablet	To reduce cholesterol **Do not drink grapefruit juice**
**Metformin 500 mg**	1 tablet		1 tablet		For diabetes **Take with meals**
**Paracetamol**	2 tablets, if needed	2 tablets, if needed	2 tablets, if needed	2 tablets, if needed	For pain. Take if needed, not more than 8 in a day

Prior to interview, written informed consent for participation in the study was obtained from all participants. Before the interview with patients, their knowledge and adherence to their prescribed medication was explored. All patients were interviewed in their own homes and were asked to produce all the prescribed and over-the-counter medication they currently had, whether they were taking it or not. They were then asked about each medicine individually to see if they knew what they were taking it for, and asked to describe exactly when and how they took them. Details were taken from the prescription labels by the interviewer as a comparison. Patient interviews focussed around exploring their experiences of managing multiple medications and any problems they encountered, where they get their information about their medication, and experiences of current services (See Additional file 2- patient interview schedule). Patients were then shown the example UMS chart (Table 2) and asked if they thought it would be useful to them, what they thought it should include, who they thought it should come from (GP or pharmacist) and any concerns they might have.

All interviews were conducted by the author (CK) and audio-recorded with consent and fully transcribed. Interviews lasted between 32 and 63 min (mean 44 min) for practitioners and between 12 and 66 min (mean 42 min) for patients. Analysis was conducted according to the constant comparative method [15], whereby analysis was carried out concurrently with data collection so that emerging issues could be iteratively explored. Development of conceptual themes was inductive. Transcripts were analysed independently and coded by hand; emerging themes were discussed until consensus was achieved, and a coding framework that included higher level themes and relevant data was assembled in Microsoft Word. Each transcript was analysed individually and then in groups, with the healthcare professional transcripts analysed separately from the patient transcripts but with comparisons made across datasets. Analysis was conducted by the main author and a sample of 10% were also coded by other team members. All team members reviewed the themes with extracted quotes to ensure agreement of themes and coding of data. Quotes are used to illustrate key themes. (*Participant codes: GP = GP, Ph = pharmacist, P = patient*).

Ethical approval was granted by NRES Committee West Midlands- Solihull (13/WM/0125).

## Results

Firstly, we looked at the perceived need for further support for these patients in terms of improving knowledge and rectifying errors or poor adherence. We also discussed perceptions around existing services and their ability to support these patients.

### The need for support in medication adherence

Using the information collected on patient knowledge and adherence to their prescribed medication, we explored the need for further support for these patients. Patient understanding of their medication was mixed, with patients demonstrating some broad knowledge about their medications, for example, the anti-angina medicine nicorandil was described as “for the heart” (P5) when it would have been more accurate to state that it was for treatment of angina. However, 11 out of the 15 patients did not know what at least one of their medications was for. Two patients used a community monitored dosage system (dosette) both of whom reported not knowing what the majority of their medication was for. Two patients had misunderstood what at least one of their medications was for (for example, the cholesterol-lowering medicine atorvastatin described as a “blood thinner” P4).

Nearly all patients reported good adherence to their medication, but when asked for specific details about how and when they took each medicine, discrepancies were noted. Three patients were not taking their medicines as described on the prescription labels, with a total of 5 medications being taken incorrectly. For example patient P10 had been prescribed the diabetic medicine metformin ‘1 tablet 3 times a day’ but was actually taking 1 tablet at breakfast and 1 at night. Four patients were choosing not to take some of their medications, or taking it sporadically (without informing their GP) and *still collecting* it on repeat prescription (P2 was being prescribed the nutritional supplement Adcal D3™ and the painkiller tramadol but had not taken them for the last 3 years). Only 3 of the 15 patients admitted to forgetting to take their medications, but during the interview nearly all patients recalled incidents where they had forgotten to take some doses and having to take it at a later time when they remembered. In summary, the majority of wrong medicine taking was unintentional with only 4/15 patients reporting intentional non-adherence that had not been discussed with either a GP or pharmacist.

The interviews explored GP and pharmacist perceptions on how well patients manage their medication regimens. Most practitioners felt that some patients experienced difficulties with medication management. It was also felt by most that this would therefore impact on adherence:

Not as many people as I’d like to, understand why they should take what they take, so I’d say more than half don’t. And, I think the fact that they don’t then does make a big difference to their adherence. GP1

I’m well aware that it’s not as clear as you would sort of assume and there is quite a lot of misinterpretation around dosing schedules and other things that they have. Again, I’d have said probably half of those patients won’t take it correctly, if not more. Ph1

### The Universal Medication Schedule (UMS)

To explore views on the UMS, patients and practitioners were given an example chart (Table 2). The interviews explored views on: usefulness/usability, how it might be delivered and feasibility of implementing it in the UK care system.

### Attitudes towards UMS

Practitioners generally expressed positive views about the UMS, with all GPs and most pharmacists thinking that it could be of benefit to particular patients:

I think the ‘what it’s for’ is a really good idea. Because I’m forever sending out patients, with their repeat prescriptions, and I’ll write on what they’re all for. GP2

Three of the pharmacists did not feel that the chart would add much to current practice. However, these views may have been affected by their perceptions around the services they provided themselves:

I don’t think it’s going to be of massive benefit in terms of that chart there where it’s all laid out, there are some sort of poorly educated patients, and I don’t think it would necessarily help all them. I think looking at that could be a bit over-facing actually. Ph1

Patients also had mixed views on how beneficial the UMS might be for them. When asked if they thought it would be useful for them, on the whole patients liked the simplicity of the chart - preferring having all the information to hand when taking their medications:

It’s short and sweet and you don’t have to sit and read through all that [PILs], because I don’t know people that read it anyway P1

Yeah, it would be, because that way I could just pin it…well, tape it in the cupboard and as I open the cupboard I can see it as I’m doing my tablets as well P10

Patients often felt that they understood and managed their medicines well and therefore did not need further support such as that provided by the UMS chart:

I know what’s in the box and I know what it’s for. Well, the information’s there so I know what it’s for, like, you know…I don’t think it would be any more helpful than what I’ve got already, to be honest with you. P7

However, as described earlier many patients, even those who thought they managed their medicines well, were not taking them exactly as prescribed and were unclear on what their medicines were for.

### How it could help

The ‘Special Information’ column providing details on what medications are for and any further instructions was thought to be particularly helpful for patients:

I think what’s helpful is so they know what they’re taking these tablets for. So that last column is very helpful. Very helpful indeed, because not only would it tell them when to take them, but it would tell them why they do need to take it. GP4

The majority of GPs thought that improved health literacy would facilitate discussions with patients about their medications and therefore help patients make informed decsions:

These special instructions, or what it’s for, that’s the bit that really matters, because once they know what it’s for, then they can start to challenge and question, and say, “well actually you’ve given me this, and you say it’s for my cholesterol, but I thought my cholesterol was alright doctor?” GP3

However, this seemed to raise an issue for some GPs who expressed that improved understanding might not always be beneficial to adherence:

There’s one danger though, and I think this probably refers again to my specific community here, some patients may take the decision as to whether they want to take a medication or not into their own hands, because they know that this is for blood pressure, they may say, well, today I feel really well, I’ve checked my blood pressure on my home machine today and it was normal, so I probably don’t need that tablet. GP8

Although this view was only expressed by a couple of GPs this may have implications for delivery of the UMS.

Practitioners also identified that the UMS could be a useful tool for communicating between settings so between general practice, pharmacy, hospital and the patient, with everyone having access to the same information. One pharmacist who also worked in a hospital pharmacy stated that:

With my hospital hat on it’s really useful to have a list of all the medication the patient is on in case they get admitted and they can take it with them. Because when we used to do the medicines reconciliation it was a nightmare, they’d come in with bags and bits and drabs and stuff and you wouldn’t know what they were on. Ph1

GPs also thought that consistency throughout the service was a key issue for patient care:

This will allow consistency of message from us as well as from the pharmacist, and I think it’s the consistency that’s probably more important than anything else. GP3

Patients thought that the UMS would be of use but were less clear in how it would help them. Unlike the practitioners interviewed, who did not identify that they thought it would reduce errors, some patients thought that it would help them prevent errors:

Oh that would be a good idea that…Because I get that mixed up sometimes, you know, and I think, oh God, and then I look through the tablets and think, oh, I’ve got two of them, oh, I’ve got three of them, so the one that I’ve put extra I throw away! P10

Some patients thought that the UMS alone would not be sufficient to help them because it doesn’t act as a reminder to take the medication or provide any visual evidence (such as an empty container) that they have taken their medications that day. However, it was suggested that if used in conjunction with a self-filled dosette box, it may improve adherence to medicines. One patient who raised this used her own self-filled dosette box to illustrate:

But, as I say, just something in print isn’t as good as the physical thing of having the tablets there [in a dosette tray] P9

### Delivering the UMS

When asked who they thought should deliver the UMS to the patient, there was evidence of role conflict. The majority of GPs thought that in terms of time and practicalities, it would be best delivered in pharmacies. Conversely some pharmacists thought the chart would be best delivered by the GP.

However, most GPs and pharmacists stated that it would have more impact if delivered by multiple sources:

So hit it home at discharge [from hospital] and then the GP can do that but so can the pharmacist, we can all sing off the same message but at different points of the process, and if somebody’s missed at one there’s a likelihood to be caught in the net later on. Ph4

Suggestions were made as to how the UMS chart could be best implemented. There are a number of GP and pharmacy services already in place in the UK (See Additional file 3- The Community Pharmacy Contractual Framework (CPCF)), with the aim of improving knowledge and supporting medicines adherence in patients. However, these were not always well perceived. Integrating the UMS into the existing services to provide enhanced support seemed to be logical, and suggestions were made for linking it to a number of these services:

### GP system

In general practice, consultation time and medicines reviews are the main vehicle to support medication use in patients, but limitations with this system were acknowledged:

Because they often…they’ll have some [medications] and then get more added and more added and it’s…you might tell them what the new one is but then it’s reminding them again of what the older ones were and it can get…I’m sure it gets very complicated. GP5

Some GPs suggested that a tool which can automatically add new medicines on to an existing chart could help support this process:

So, what would be nice, is that something like this [UMS] sits allied to the GP prescribing element, it recognises everything that’s on repeat prescription, you press a button, and out it comes. GP3

### Medicine Use Reviews (MUR)

MURs are an incentivised scheme delivered by pharmacists. Time pressures and targets to complete the maximum number of reviews per year (400) were perceived to have a negative impact on the value of MURs and on targeting the right patients. As a result of this, most GPs stated that they saw little value to MURs and that they viewed them as ‘tick box’ schemes that rarely raised important issues:

I think the Medicines Use Reviews were entirely without purpose and benefit and should have been not done, and we in fact didn’t even read the stuff the pharmacist sent us we used to throw it straight in the bin. GP1

Patients who had been offered a MUR were generally positive about the service although only 4 had actually taken part in a review. It was thought by most practitioners that this would be a good place to introduce the UMS as it would allow time for discussion. However, issues around targeting and the small numbers of patients receiving these services would remain.

I think talking through it would be quite a good process, it is quite a good lead into an MUR, to be quite honest with you, or a medication review with the doctor…I wouldn’t just have it as a bag stuffer, I think people sometimes ignore the things that are just stuffed in the bag. Ph4

### Monitored Dosage System (dosettes)

The dosette system provides selected patients with their medications already divided into daily, timed doses. GPs and pharmacists reported both benefits and problems associated with using the MDS/dosette system. Two patients in the sample were currently using the MDS system and both had very poor knowledge of their medications. However, they both felt that it had improved their adherence to some degree:

I tended to take them all at once rather than splitting it up. And it’s only because they’ve been split up on this system [dosette] that I started following that, to some extent, P3

Dosette boxes do have a list of the medicines included but would not usually indicate what health conditions the medicines are for. It was also commented on that this information was not presented well and would be difficult for some patients to read. The potential for including the chart in the dosette tray was suggested:

Well, very often with the [dosette] pack, you know, when you lift the lid it has that written inside, doesn’t it? I must say it’s not very well presented in some of them. It’s like it’s come off a computer from the 80’s or something, but at least it’s there. You could upgrade that. GP7

Again the majority commented that introducing the UMS throughout the system from secondary care to primary care creating more consistency and improving patient care would be the most efficient use of the chart:

What we want, and I think what’s really important for patients, is that if you have the same system all the way through, because that’s where confusion comes in otherwise, you know, different formats? So, it would be really nice, because we’re always talking about integrated care these days, and lack of fragmentation of care, and having a consistent message. Well, this is one of those examples. It should be adopted right the way through. GP3

### Feasibility of implementing UMS

All practitioners thought that the chart would have to be linked into existing computer packages and to be self-populating, otherwise it would place too much strain on GP or pharmacist time:

Otherwise it won’t get done, you know, there just isn’t time in consultation to be producing something like this. So it needs to be, sort of, effortless to produce. But you could have a C side [to a prescription form] which you’d produce…you know, you’ve got your B side on your prescription, and you have a C side which are printed out like this, yeah GP6

Most practitioners agreed that the paper format was the most practical with the current population. However, issues around keeping the document up-to-date and ensuring a patient is using the correct version are a consequence of using hard copies which become historic soon after printing:

But they like paper, and the other thing about paper is that if there’s any question about it, they can pick it up, they can take it to the hospital with them, they can show it to their relatives. So, I think paper is the way forward. But, I guess my concern about paper is, is it always up to date? GP2

Another possible limitation of using hard copies is that if a patient had only one copy it is unlikely they would have it with them all of the time:

Even if you don’t lose your bit of paper, it might be upstairs when you’re downstairs, and it could be time for your medication, so it’s not going to be with you all the time is it? GP3

Thinking about feasibility of delivering the UMS chart in the future, many practitioners thought that it may need to move to a digital format. The point was also made that as the population ages more people will be used to electronic devices to support their health care:

I think as people who are like my age, you’re not going to stop using your smart phone and your computer just because you get to 65. The current generation of 65 to 75 year olds probably don’t use computers much although I do come across patients that do. In the future, if you’re going to plan something for the future, then there must be scope. Ph6

## Discussion

### Summary

It was felt by both GPs and pharmacists, that many patients with multimorbidity and polypharmacy need further support to help improve their understanding of and adherence to their medications. Most patients thought that further information and making the important information easily accessible, would be beneficial. Most patients felt that they managed their medication well. However, all except one patient had aspects of their regimen that they did not understand or that they did not adhere to correctly. Intentional and unintentional non-adherence was seen.

Although potential limitations were identified, most practitioners and patients thought that the last column of the chart (Table 2), which gives clear information on what the medication is for and any specific directions (such as take with food), was particularly useful for improving health literacy. Most practitioners thought that improving health literacy would help support patient choice. However, a small number of GPs displayed paternalistic tendencies and thought that improved health literacy might not always be beneficial to adherence. None of the practitioners mentioned that they thought it would reduce errors in medicine-taking, although this was not asked as a specific question.

As well as helping to improve patients’ understanding, it was also felt by practitioners that the UMS could be used as an effective tool to aid communication between the different services involved in a patient’s care and also as an aid for carers in supporting/managing another person’s medication. Although initially conflicted about who should deliver the UMS, practitioners presented a number of existing services where they thought the UMS could easily be integrated and that it could be used across services to provide a consistent message to patients. However, there was evidence of a lack of willingness to take responsibility for including the chart in a patient’s care, which could potentially be a barrier to use. With the identification of suitable existing services the UMS could be incorporated into - and the proviso that the chart would need to be self-populating to some degree - most GPs and pharmacists thought that providing patients with a UMS chart could be feasible, acceptable and beneficial to patients.

### Previous literature

Our results on patient understanding of their medicines supports other research which has reported that less than a quarter of people on multiple drug regimens knew the names and purposes of all their medicines [12,13,16]. There is little research published on practitioner and patient views around the introduction of reminder charts for support in medication adherence. Early research conducted in 1993 in the UK, on the introduction of a reminder chart, demonstrated significant results [13] but has not been taken forward in the UK. The research by Raynor and colleagues focussed on a different population to that used in this research. In the Raynor study (1993), patients may have had single conditions (rates of comorbidity were not reported), and they also used a lower threshold for polypharmacy (maximum of 6 medicines). In contrast the present research only included patients with 2 or more long-term conditions who had to be taking at least 5 medications with no upper limit. We are therefore looking at patients with a higher level of complexity and burden caused by treatment regimens. US research on the UMS has also shown positive results, but much of the published literature is focussed around labelling changes as well as the simplification of medicine regimens in to a chart. The authors report that the UMS has demonstrated efficacy and effectiveness on comprehension, consolidation of regimens and improved adherence measured by pill count [12]. However, the research in the US does not measure patient or provider acceptability, or the impact of administering the UMS chart alone, independent of changes made to labelling instructions. One of the key perceived benefits to introducing the UMS was that it could be implemented across healthcare settings. The involvement of multiple services in a patients care has been shown to carry an increased risk of fragmented care and frequent failures in communication [17]. The need for less fragmented, minimally disruptive care for patients with multiple conditions has been commented on in much of the recent literature around multimorbidity [18-20]. The UMS, if introduced to hospital pharmacy, primary care and community pharmacy could provide a common vehicle and language to ensure that all of these services are working in a ‘seamless’ way when it comes to prescribing and explaining medicine regimens to patients.

One issue is whether or not improved knowledge does equate to good adherence. For those patients in this sample who were using the MDS system, their knowledge of their medication was poor but their adherence was no worse than patients who did know what the majority of their medication was for. This supports other research which shows that providing clear instructions or information does not always result in good adherence [14]. Simplification of a regimen can be helpful to some patients [21]. However, simplification alone may not solve the adherence problem [10]. Horne et al. [10] proposed that complexity is not necessarily the key issue but how well the treatment fits in with an individual patients routine, expectations and preferences.

### Strengths and limitations

One of the strengths of this research is that it explores the issue of medication adherence in patients with multimorbidity and polypharmacy, targeting a population with complex medicines regimens. Another key strength is that by interviewing patients, GPs and pharmacists the research explores medication adherence from the different perspectives of those involved in all aspects of medication from prescribing and monitoring, dispensing and advising, to self-management in adherence. As with most studies that rely on volunteers to take part in research, the participants may not be fully representative of their populations. Some practitioners also stated that they had trouble recruiting those patients who were particularly poor at adherence to their medication, although we found evidence of clear adherence issues in the population that did participate. This research raised some questions about current provider behaviours but this was not the focus of the interviews. There is potentially the need for further research on changing prescriber and provider behaviours to better support medicines use in patients. This research does not provide evidence as to whether the UMS will actually improve adherence and a trial is needed to assess this. However, as previously stated, other research in this area has shown significant improvement in both health literacy and adherence with the use of similar tools.

### Implications

The UMS may impact on unintentional non-adherence by simplifying a patients’ medicines regimen, thereby reducing burden, and also in acting as a reminder chart. It was generally felt that intentional non-adherence is more difficult to address but it is thought that by increasing a patients’ knowledge and health literacy, which may not necessarily improve adherence, then patients can at least make informed decisions about their medication. However, not all GPs agreed that this would lead to appropriate decisions by patients. Practitioners held mixed views, and although a number of factors would need to be addressed for successful use in primary care, it was generally thought that implementation of such a chart could be feasible and potentially beneficial. Many of the GPs and pharmacists interviewed suggested that the use of a uniform tool such as the UMS, used throughout a patients care, could help reduce fragmentation if the patient is receiving the same message in the same format from multiple sources. It was also suggested that the use of a single tool could help communication and medicines reconciliation between primary and secondary care and community pharmacy services. However, one major obstacle to this is the numerous different IT systems currently in use and the lack of a single patient record. The design of a digital ‘app’ which patients could access on their phones, computers or tablets and which could be accessed and updated by health professionals may be a future consideration.

## Conclusions

One of the key benefits of using the UMS in practice, as identified by GPs and pharmacists is that it could be introduced across services. In this way it may aid in medicines reconciliation between healthcare settings to ensure continuity of message and improve patient experience and create more joined up working between services. Views around the UMS and its ability to improve adherence were mixed. However, it was felt that it could be of benefit to particular patients and could be easily placed within existing services and practices.

Further research is needed to test implementation in different services and to assess outcomes on patient understanding and adherence.

Participant codes-

- GP- General Practitioner
- Ph- Pharmacist
- P- Patient

## Additional files

Additional file 1:
**GP/Pharmacist interview schedule.**
Additional file 2:
**Patient interview schedule.**
Additional file 3:
**The Community Pharmacy Contractual Framework.**

## Abbreviations

GM CLAHRC: Greater Manchester Collaboration for Leadership in Applied Health Research and Care
GMCLRN: Greater Manchester Comprehensive Local Research Network
GP: General Practitioner
MDS: Monitored Dosage System or dosette
MUR: Medicines Use Review
NHS: National Health Service UK
PPI: Patient and Public Involvement in research
PRIMER: Primary Care Research in Manchester Engagement Resource
UMS: Universal Medication Schedule
