# Supplementary material for: The potential for using a Universal Medication Schedule (UMS) to improve adherence in patients taking multiple medications in the UK: a qualitative evaluation
Source: BMC Health Serv Res. 2015 Mar 11;15:94. doi: 10.1186/s12913-015-0749-8 (PMC4359545; doi:10.1186/s12913-015-0749-8)
Supplement: Additional file 1: — GP/Pharmacist interview schedule. [file 12913_2015_749_MOESM1_ESM.doc]

**Additional file 1:GP/ pharmacist interview schedule v3- 28/06/13**

**Medication adherence**

- How well do you think most patients are able to *understand* their medicine regimens?
- How well do you think most patients are able to *adhere* to their medicine regimens?
- Do you ask patients if they have anyone to help/ have any problems with taking their meds?
- Do you think patients always tell you about problems/ recognise that they are having problems?
- Do you think polypharmacy raises specific problems for patients in terms of medication adherence? If so what?
- What do you think are the personal issues in poor adherence?
- Any examples of ‘trade-offs’ made by patients regarding the relative importance of medicines for certain conditions (e.g. symptomatic treatment vs prevention/non-symptomatic)
- Adjustable dosage meds (eg warfarin, insulin, pain meds). How well do patients tend to manage these medications?
- How do you think changes in brand/appearance of medicines effects patients? Do you explain changes when prescriptions are collected?

**Reviewing medications**

***[GPs only]***

- What experience do you have of doing medication reviews with these patients? Could you talk me through what you did in the last medicine review that you did?
  - When do you tend to do reviews? *(prompts- requested by patient, as part of disease monitoring – QOF, periodically, after SAE/hospitalisation, to meet pharmacy targets)*
  - What format do the reviews usually take – with or without patient present?
- Medication reviews has recently been removed from the QOF guidelines.
- What do you think about this?
  - What if any effect do you think it will have on how you practice?
- What do you think about pharmacists’ roles in medicine use reviews?
- How do you think this fits in with your role?
- Do you have much contact with local pharmacists in regards to medicines use reviews?
- *If there are issues how do they think these could be improved?*

***[Pharmacists only]***

- I’d like to ask about your experience of community pharmacy services like MURs.
- What experience do you have of doing MURs with these patients? Could you talk me through what you did in the last review that you did?
- What do you think of these services?
- What if any are the benefits to patients? Do they target the right people?
- Do you think they have any impact on adherence?
- What do you think should be put in place to help patients to adhere?
- How much contact do you have with local GPs in regards to MURs?
- How do you think GPs receive the care/medications suggestions you make as part of the reviews?
- Cost implications of changes (eg soluble form of tablets, pain patches etc)- what consideration is this given?
- Any issues of hierarchy/ cultural agendas?
- *If there are issues how do they think these could be improved?*

**Community Monitored Dosage Systems**

- Have you any experience of Community Monitored Dosage Systems (dosset boxes)? Could you talk me through what you do for this?
  - Who uses this service (describe pt)? Is it targeting the right patients?
  - What are the patient benefits?
  - Do you think it has an impact on adherence? –In what way?
  - Do you think there are any limitations to this system?

**Improving medication adherence**

- Thinking about patients who maybe don’t need/qualify for dosset. What do you think would help patients to correctly take their medications? Do you do anything to help particular patients? If so what do you do with them?
- Thoughts about the UMS (summary circulated to them pre-interview).
  - Had you previously heard of the Universal Medications Schedule (or reminder charts or MAR charts)?
  - What are your initial thoughts on UMS?
  - How would you suggest ‘as needed’ meds/ variable dose meds/ over-the-counter meds/ supplements are added to charts? Do you think it is helpful to include all of these?
  - What would be the most useful for you/ for patients?
  - How feasible do you think it is to implement UMS?
  - Do you think it should just be used with certain patients? Who would you target it to? Who wouldn’t it work for?
  - What proportion of patients do you think it could be useful for?
  - At what point should it be implemented (with the introduction of a new medicine to an existing regime/ after hospital admissions/ when a patient next refills their meds)?
  - Do you think it should be given by the GP (computer generated from patient records) or by the pharmacist (pharmacy records)? Does it matter?
