# Supplementary material for: The potential for using a Universal Medication Schedule (UMS) to improve adherence in patients taking multiple medications in the UK: a qualitative evaluation
Source: BMC Health Serv Res. 2015 Mar 11;15:94. doi: 10.1186/s12913-015-0749-8 (PMC4359545; doi:10.1186/s12913-015-0749-8)
Supplement: Additional file 2: — Patient interview schedule. [file 12913_2015_749_MOESM2_ESM.doc]

**Additional file 2: Patient Interview schedule v2- 25/04/13**

**Thinking about your medications:**

- Have you ever had any difficulties or problems with following the directions on the packaging?

(If yes, what did you do- go back to pharmacy/GP, stop taking medicine?)

- Have you ever had any serious problems (worsening of condition, emergency care needed) due to your medications? Were medicines changed as a result?
- Talk me through a day with your medicines. How many separate times in a day do you have to take a dose of medicine?

Does anyone else help you with the taking of your medications (spouse/carer/community support services)?

- Are there any treatments you receive such as injections or specific medicines given by district nurses or home care nurses? How does this affect how you take your other meds, if at all?
- Do you think some of your medicines are more important than others?

(If so, which ones and why?)

- Do you sometimes not take all/some of your medicines? If so, why?
  - - Choose not to- what led to you making this choice?
    - Forget to take them- does anyone help you, do you have any kind of system to help you remember?
    - Forgot to order/get your repeat prescription?
    - Is it expensive, do you need to pay for your prescriptions?

**Knowledge about medications:**

Contribution of different health professionals to their medicines knowledge and use

- Who have you discussed your medication with?
- Does your GP or pharmacist know about everything you use, or choose not to use? Have you ever discussed this with them?
- Who do you tend to take advice from about your medicines (GP/pharmacist/other)
  - Did anyone explain your medications to you when you first got them? What did they tell you?
  - Have you ever had to ask for further information on how to take your medication?
    - Where did you go for that information?
    - What did you get? Patient leaflet/ internet site/review with practitioner (GP or pharmacist)
    - How useful was the information that you were given?
  - Have you ever had a sit-down review of all your medication?

If so, who was it with? Was it useful? What did you do/discuss in it?

**Improving medication adherence**

- What helps you remember to take your medications (existing strategies)?
- Are some easier than others to remember? If so, which and why? (eg symptomatic conditions)
- Do you think you need help with your medications to make sure you are taking them correctly?
- Thoughts about the Universal Medication Schedule *[given an example chart].*
  - What do you think of this chart in terms of supporting your medicines taking?
  - Would this be useful for you?
  - What would you want included on it- just prescription medicines, other over-counter medicines, anything else?
  - Would this be useful for other people? In what circumstances?
