# Supplementary material for: The potential for using a Universal Medication Schedule (UMS) to improve adherence in patients taking multiple medications in the UK: a qualitative evaluation
Source: BMC Health Serv Res. 2015 Mar 11;15:94. doi: 10.1186/s12913-015-0749-8 (PMC4359545; doi:10.1186/s12913-015-0749-8)
Supplement: Additional file 3: — The Community Pharmacy Contractual Framework. [file 12913_2015_749_MOESM3_ESM.doc]

**Additional file 3: Box 1 The Community Pharmacy Contractual Framework (CPCF) is made up of three different service types:**

**Essential services (e.g. Repeat dispensing, Signposting to other services, Self-care support):**

Repeat dispensing- enables GPs to issue a single prescription for up to a year, which pharmacists are then able to dispense in instalments.

Signposting-Pharmacies are expected to help people who ask for assistance by directing them to the most appropriate source of help.

Self-care support- to help manage minor ailments and common conditions, by the provision of advice and where appropriate, the sale of medicines, including dealing with referrals from NHS Direct/NHS 111

**Locally Commissioned Services (e.g. Condition-specific services for asthma, weight loss and diabetes, Clinical medication review):**

 LPCs and contractors are able to negotiate to provide services in accordance with these specifications where a local need for the service is determined. Alternatively LPCs, contractors or the service commissioner are free to develop their own local services in response to identified needs

**Advanced Services (e.g. Medicines Use Review and Prescription Interventions, New Medicine Service):**

MURs and prescription interventions- introduced in 2005, as the first advanced service to be provided by accredited pharmacists in accredited pharmacies. During an MUR, the pharmacist has a structured discussion with the patient about the use of their medicines.

The New Medicine Service (NMS) introduced in October 2011, for newly prescribed medicines for a small number of conditions, namely asthma and COPD, type 2 diabetes, anticoagulant/anti-platelet therapy and medicines for hypertension. Pharmacists providing the service follow up new prescriptions for these long-term conditions with interventions designed to reinforce key messages about medicines use and resolve patients’ queries and concerns once treatment has started.
